# Temporal patterns of visitation of birds and mammals at mineral licks in the Peruvian Amazon

**DOI:** 10.1002/ece3.7006

**Published:** 2020-11-11

**Authors:** Brian M. Griffiths, Mark Bowler, Michael P. Gilmore, David Luther

**Affiliations:** ^1^ Department of Environmental Science and Policy George Mason University Fairfax VA USA; ^2^ School of Science, Technology and Engineering University of Suffolk Ipswich UK; ^3^ Suffolk Sustainability Institute Ipswich UK; ^4^ School of Integrative Studies George Mason University Fairfax VA USA; ^5^ Department of Biology and Smithsonian Mason School of Conservation George Mason University Fairfax VA USA

## Abstract

Mineral licks are key ecological resources for many species of birds and mammals in Amazonia, providing essential dietary nutrients and clays, yet little is known about which species visit and their behaviors at the mineral licks. Studying visitation and behavior at mineral licks can provide insight into the lives of otherwise secretive and elusive species. We assessed which species visited mineral licks, when they visited, and whether visits and the probability of recording groups at mineral licks were seasonal or related to the lunar cycle. We camera trapped at 52 mineral licks in the northeastern Peruvian Amazon and detected 20 mammal and 13 bird species over 6,255 camera nights. Generalized linear models assessed visitation patterns and records of groups in association with seasonality and the lunar cycle. We report nocturnal curassows (*Nothocrax urumutum*) visiting mineral licks for the first time. We found seasonal trends in visitation for the black agouti (*Dasyprocta fuliginosa*), red howler monkey (*Alouatta seniculus*), blue‐throated piping guan (*Pipile cumanensis*), red brocket deer (*Mazama americana*), collared peccary (*Pecari tajacu*), and tapir (*Tapirus terrestris*). Lunar trends in visitation occurred for the paca (*Cuniculus paca*), Brazilian porcupine (*Coendou prehensilis*), and red brocket deer. The probability of recording groups (>1 individual) at mineral licks was seasonal and related to lunar brightness for tapir. Overall, our results provide important context for how elusive species of birds and mammals interact with these key ecological resources on a landscape scale. The ecological importance of mineral licks for these species can provide context to seasonal changes in species occupancy and movement.

## Graphical abstract

Many species of animals in the Amazon visit mineral licks, consuming soil to supplement their diet. Using camera trap data, we show that visits to mineral licks are seasonal for many species. These trends in visitation are likely due to species‐specific factors such as reproduction, predator avoidance, seasonal diet shifts, and resource use.

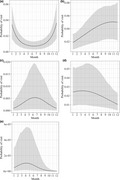

## 1 INTRODUCTION

Tropical forests, particularly the Amazon rainforest of South America, have the highest terrestrial biodiversity (Brown, 2014; Schipper et al., 2008) and primary productivity (Beer et al., 2010) in the world. Many of the species in the Amazon are secretive and elusive, and little is known about their ecology and behavior relative to similar species in temperate latitudes. In particular, little is known about the activity patterns, ranges, and social structure of many of the large mammals and birds in the Amazon. However, it is known that many frugivorous and folivorous mammals and birds visit key ecological sites called mineral licks (e.g., Blake et al., 2010, 2011, 2013; Link et al., 2011; Tobler et al., 2009), which provides a unique opportunity to study the behaviors of these otherwise elusive species.

Mineral licks are naturally occurring sites in the forest where animals visit to consume soil, a behavior known as geophagy (Abrahams & Parsons, 1996; Panichev et al., 2013). These sites generally occur where outcroppings of geologic materials have been exposed to erosion (Klaus et al., 1998; Lee et al., 2010). Mineral licks in the Amazon frequently occur in *terra firme* forests and along riverbanks. They are visited by a diverse array of species, including large‐bodied mammals such as the Brazilian tapir (*Tapirus terrestris*) and red brocket deer (*Mazama americana*), rodents such as the paca (*Cuniculus paca*) and black agouti (*Dasyprocta fuliginosa*), and arboreal mammals such as the red howler monkey (*Alouatta seniculus*) and Brazilian porcupine (*Coendou prehensilis*) (Blake et al., 2011; Molina et al., 2014; Montenegro, 1998, 2004; Tobler, 2008; Tobler et al., 2009). Mineral licks are also visited by parrots, pigeons, and large‐bodied bird species such as the blue‐throated piping guan (*Pipile cumanensis*) and Spix's guan (*Penelope jacquacu*) (Montenegro, 2004). Congregations and relatively high levels of activity at specific locations such as mineral licks tend to attract predators, such as jaguars (*Panthera onca*) (Matsuda & Izawa, 2008) and ocelots (*Leopardus pardalis*) (Griffiths et al., 2020), which can cause species to be especially vigilant when they are at mineral licks (Link et al., 2011).

The drivers behind geophagy likely vary among species and mineral licks. For example, in the Amazon, many mammal and bird species visit mineral licks to obtain essential nutrients that are missing in their diet (Matsubayashi et al., 2007). Amazonian parrots visit mineral licks to obtain minerals such as sodium (Brightsmith et al., 2008; Lee et al., 2010). Amazonian bats, particularly female bats that are pregnant (Bravo et al., 2008), seek minerals such as sodium, potassium, and magnesium (Ghanem et al., 2013). Studies focused on other species and regions suggest a different driver of geophagy: the consumption of clays that aid in relief of gastrointestinal ailments (Kreulen, 1985; Mahaney et al., 1997), such as chimpanzees in Africa (Mahaney et al., 1996) and several bird species of New Guinea (Diamond et al., 1999).

While mineral licks are “hotspots” of diversity in lowland Amazonia (Blake et al., 2011) and visits to mineral licks are of great importance for many species in Amazonia (e.g., Blake et al., 2010; Tobler, 2008; Voigt et al., 2008), the factors associated with their visitation rates, sociality, and the timing of their visits are vital for a more holistic understanding of their ecology. For example, mineral licks are thought to be key locations for social interactions among animals including aggression in moose (Couturier & Barrette, 1988) and white‐tailed deer (Weeks, 1978) and communication through urine deposition in tapirs (Montenegro, 2004). Observations at mineral licks can also provide insight into vigilance behavior of these animals as visits to mineral licks can leave animals exposed and vulnerable to predation (Parrots: Brightsmith & Villalobos, 2011; Primates: Link et al., 2011). Visitation rates and behaviors at mineral licks could also be affected by environmental variables, such as the lunar cycle and seasonality (e.g., Blake et al., 2010 for increased mineral lick use in the dry season by red howler monkeys). More information about the environmental factors and behaviors associated with mineral lick visitations could help determine animal territory size and quality, and movement throughout their territories and across the territories of other individuals (e.g., tapir movement, Tobler, 2008).

Rates of visitation, activity patterns, and other behavioral analyses can provide a useful window into the ecology of many understudied species of mammals and birds and expand our knowledge of the roles that mineral licks play in the ecology of these animals. Here, we assess the activity patterns and environmental variables associated with the visitation and number of individuals recorded of medium‐ and large‐bodied mammals and terrestrial birds at a relatively large network of mineral licks in the northeastern Peruvian Amazon. In this paper, we investigate the following research questions:

1. Which animals visit mineral licks, and how frequently?
2. What are the activity patterns of species that frequent mineral licks?
3. Are visitation patterns of animals at mineral licks associated with abiotic environmental factors such as season or lunar cycle?

## 2 MATERIALS AND METHODS

### 2.1 Study site

**FIGURE 1:**
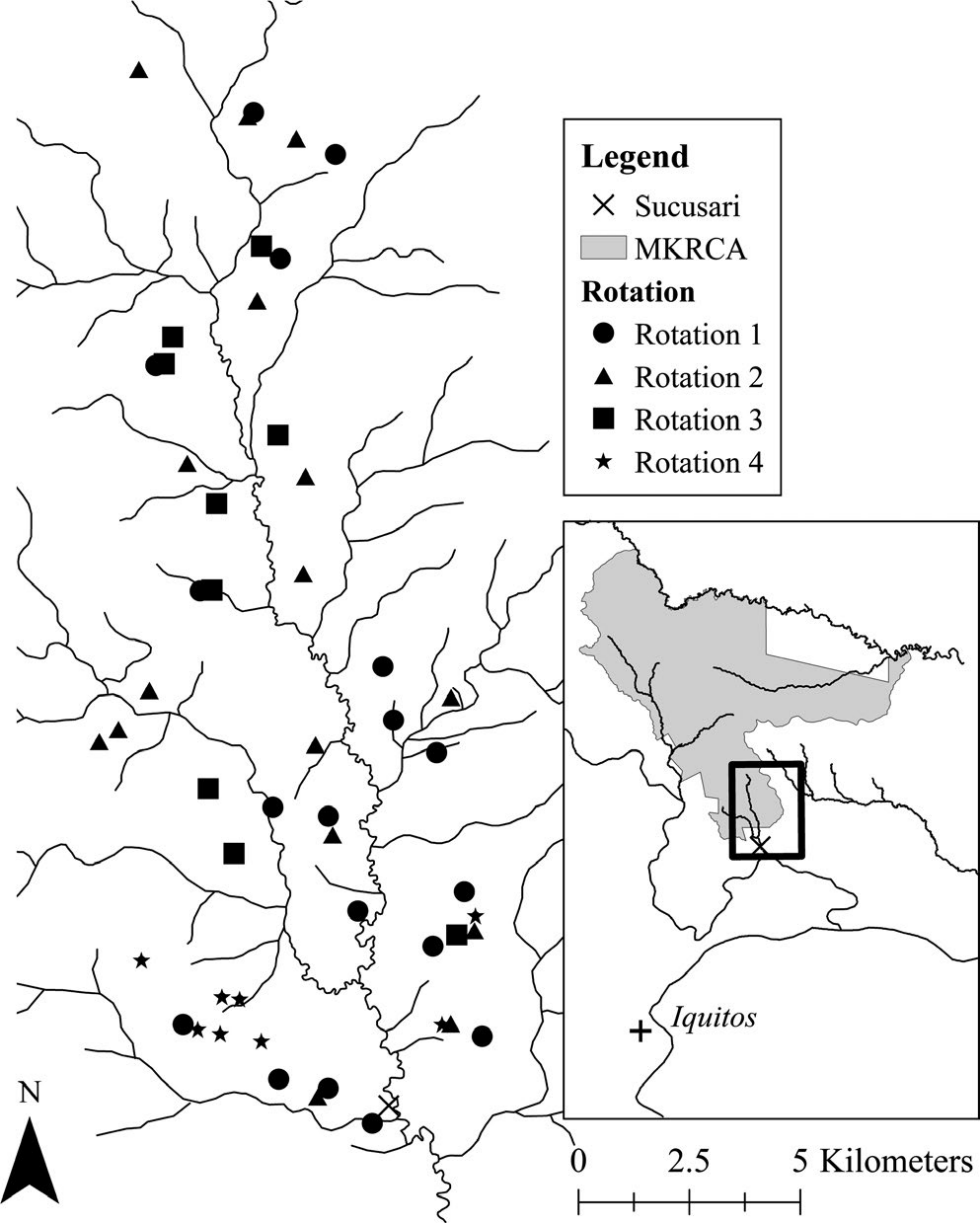
Map of camera trap locations and rotation numbers at 52 mineral licks in the study site, the Maijuna community of Sucusari and the southern portion of the Maijuna‐Kichwa Regional Conservation Area (MKRCA) in the northeastern Peruvian Amazon

Fieldwork took place in the Maijuna community of Sucusari and the Maijuna‐Kichwa Regional Conservation Area (MKRCA), a 391,039‐hectare protected area in Loreto, Peru (El Peruano, 2015). This area is about 120 km north by river of Iquitos, Peru (Figure 1). The title lands of the Maijuna community encompass 4,771 hectares and directly adjoin the MKRCA to the south. The Sucusari River is a tributary of the Napo River and terrestrial habitats include both upland *terra firme* primary rainforest and floodplain forest. The mean annual temperature is 26°C and an average precipitation of 3,100 mm per year (Marengo, 1998). The wet season consists of the months November to May, while the dry season is mainly June to October in the Iquitos region (Espinoza Villar et al., 2009).

### 2.2 Camera trapping

We installed motion‐activated camera traps (Bushnell Aggressor, Boly Scout Guard) in the Sucusari River Basin at a sample of 52 mineral licks that were identified with the assistance of Maijuna hunters. Starting in August 2018, we visited all mineral licks, obtained GPS coordinates, and placed camera traps in a series of four deployments, each lasting at least 60 days to achieve even coverage of the whole basin (Figure 1). We left camera traps undisturbed at mineral licks for the entire rotation period. Every 60 days cameras were removed, batteries and *SD* cards changed, and cameras were rotated to new mineral licks (Kays et al., 2020). During the third rotation, most cameras went to previously unvisited mineral licks, but some went to mineral licks that held a camera in August but experienced camera malfunctions that prohibited the camera from gathering 60 camera nights of data.

The mineral licks in the Sucusari River basin are generally characterized by waterlogged mud with standing water and a face, which was often associated with a slope. The area inside the lick was generally devoid of vegetation. The number of camera traps placed in each mineral lick was determined by the size and shape of the mineral lick, with the goal of recording all animal visits to the mineral lick and meeting the assumption of perfect detection (all medium‐ and large‐bodied animals entering the lick are captured). We set cameras to record three rapid‐fire images at each motion trigger with a delay of 2 min between each set of images to avoid expending the camera's batteries. Cameras were set at a minimum of 50 cm from the ground, facing the active face and entrance to the mineral lick, following Tobler et al. (2009). We determined the location of the face from signs of animal activity. Camera traps at mineral licks that did not have a face were placed facing mud with signs of active animal activity.

We identified all medium‐ and large‐sized mammal and bird species (weight > 1 kg) in camera trap images (Blake, 1977; Emmons & Feer, 1997), removed empty images, and organized data for analyses using CameraBase v1.7 (Tobler, 2015). The number of individuals and species identity in instances where multiple individuals appeared in the same photograph was also recorded. Small‐bodied birds and mammals, including bats, were removed from analyses because they could rarely be identified to species level. Mixed species flocks of parakeets were also not considered for analysis since they commonly visited in groups of several hundred individuals and could not be reliably identified to species level. Images were sorted into independent visitation events, where multiple visits by the same species within 1 hr of each other were considered one visitation event, following Tobler et al. (2008).

### 2.3 Data analysis

To assess visitation at mineral licks, we assessed the visit frequency and group size of medium‐ and large‐bodied birds and mammals at mineral licks. We calculated the mean visit frequency for terrestrial bird and mammal species which were recorded at least ten times during the study period. Mean visit frequency was calculated as the number of independent visitation events per night of camera trapping for those mineral licks where the species visited at least one time. Not all mineral licks were considered in visit frequency calculations under the assumption that not all mineral licks are active for each species at all times, due to changes in occupancy, diet shifts, or reproductive periods.

The density distribution of activity time at mineral licks was calculated for all medium‐ and large‐bodied bird and mammal species which were recorded at least ten times and fifty times, respectively. The hour of day of the first image in each visitation event was used as the hour of activity for each event. We created kernel density plots of activity patterns using the *densityPlot* function in the *overlap* package (Ridout & Linkie, 2009) in R, version 3.6.1 (R Core Team, 2019).

To assess the association between environmental factors, such as seasonality and lunar cycles, with visits to mineral licks we constructed a series of generalized linear mixed‐effects models with a binomial distribution to assess whether visitation at mineral licks for mammals and birds was seasonal or related to the lunar cycle. We included only species which visited mineral licks over 50 times which had a large enough sample size to model. We used each day the camera traps were active at each mineral lick as samples (*n* = 4,645). For example, if cameras were active at 10 mineral licks on 10 August 2018, then that date was recorded in 10 different samples, each at a different lick. For each day, a 1 was recorded if the species visited that mineral lick, and a 0 recorded if it did not visit. Visitation was used as the binary response variable, and the covariates included were the month of the visit, the size of the lick in m^2^, the lick type (face present or not present), elevation in m, slope in degrees, distance the closest river or stream in m, distance from the closest hunting camp in m (a proxy for hunting pressure, see Griffiths, 2020), and the brightness of the moon calculated using the *lunar.illumination* function in the *lunar* package (Lazaridis, 2014) in R. For species that exhibited purely diurnal activity patterns, brightness of the moon was not included as a covariate in the model. Month was put in polynomial form in the model, due to its cyclical, nonlinear nature. The name of the lick was included as a random effect in the models to account for pseudoreplication. All continuous covariates were scaled and tested for collinearity before including them, with a cutoff of 0.60 (Dormann et al., 2013), variograms were visually examined to check for spatial autocorrelation, and full models were tested for overdispersion. Models were selected using a backwards‐stepwise procedure under the information‐theoretic framework (Burnham & Anderson, 2002), comparing Akaike information criteria (AIC) values to select the optimal model.

A series of generalized linear mixed‐effects models with a binomial distribution were constructed to assess whether the probability of recording groups of each species, except for the collared peccary (*Pecari tajacu*) and red howler monkey (*Alouatta seniculus*), was seasonal or related to the lunar cycle. The collared peccary and red howler monkey were analyzed separately because they commonly travel in groups larger than two individuals. Each visit to a mineral lick was considered a sample (sample sizes for each species shown in Table 1). If the minimum size of the group (the maximum number of individuals recorded in a single photo) visiting the mineral lick was greater than one individual, the response was coded as 1, and visits by individual animals were coded as 0. We used the same aforementioned covariates for this series of models, including lick name as a random effect. As above, we checked full models for overdispersion and employed a backwards‐stepwise selection approach (Burnham & Anderson, 2002).

**Table 1:** Visit frequencies at 52 mineral licks for all identified bird and mammal species which were recorded at least ten times during the study period

Scientific name	Common name	Local name	Visitation events	Percent (#) of licks visited	Mean (CI) vis. freq.
Mammals					
*Mazama americana*	Red Brocket Deer	*Venado Colorado*	1,781	88.46 (46)	103.20 (0–242.40)
*Cuniculus paca*	Paca	*Majás*	932	69.23 (36)	60.17 (8.67–111.68)
*Dasyprocta fuliginosa*	Black Agouti	*Añuje*	873	71.15 (37)	56.00 (0–130.47)
*Coendou prehensilis*	Brazilian Porcupine	*Cashacushillo*	629	61.54 (32)	43.56 (0–91.00)
*Pecari tajacu*	Collard Peccary	*Sajino*	412	63.46 (33)	37.74 (0–93.32)
*Tapirus terrestris*	Brazilian Tapir	*Sachavaca*	386	59.62 (31)	35.54 (0–79.83)
*Alouatta seniculus*	Red Howler Monkey	*Coto Mono*	124	30.77 (16)	15.79 (1.43–30.15)
*Dasypus novemcinctus*	Nine‐Banded Armadillo	*Carachupa*	30	23.08 (12)	3.22 (0–6.99)
*Mazama gouazoubira*	Gray Brocket Deer	*Venado Ceniza*	20	9.62 (5)	12.98 (0–27.73)
*Choloepus didactylus*	Linnaeus's Two‐Toed Sloth	*Pelejo Colorado*	20	9.62 (5)	12.17 (0–26.01)
*Nasua nasua*	South American Coati	*Achuni*	17	15.38 (8)	2.02 (0.57–3.48)
*Procyon cancrivorus*	Crab‐Eating Raccoon	*Achuni Grande*	16	25.00 (13)	1.71 (0–3.72)
Birds					
*Pipile cumanensis*	Blue‐Throated Piping Guan	*Pava*	116	7.69 (4)	20.35 (0–41.79)
*Leptotila rufaxilla*	Gray‐Fronted Dove	*Paloma*	113	15.38 (8)	23.16 (0–95.94)
*Patagioenas cayennensis*	Pale‐Vented Pigeon	*Paloma*	90	21.15 (11)	11.20 (0–35.58)
*Psophia crepitans*	Gray‐Winged Trumpeter	*Trompetero*	36	15.38 (8)	4.59 (0–18.78)
*Nothocrax urumutum*	Nocturnal Curassow	*Montete*	24	19.23 (10)	3.32 (0–8.60)
*Penelope jacquacu*	Spix's Guan	*Pucacunga*	23	21.15 (11)	2.27 (0–7.05)
*Aramides cajaneus*	Gray‐Necked Wood Rail	*Rascón Montés de Cuello Gris*	22	7.69 (4)	7.18 (0–24.63)
*Patagioenas subvinacea*	Ruddy Pigeon	*Paloma*	15	1.92 (1)	15.46 (NA)

Note

Visit frequencies calculated as the number of visits per 100 camera nights at mineral licks where the species visited at least once.

To analyze the probability of recording groups of the red howler monkey and collared peccary, we constructed generalized linear mixed‐effects models with a Poisson distribution and the same covariates as above. In this case, the response variable was the number of individuals in the photo with the maximum number of individuals (set as the minimum group size, as there could have been more individuals off camera) and each visit to a mineral lick was a sample. Full models were constructed and tested for overdispersion. We proceeded with the model selection process as described above.

All generalized linear mixed‐effects models were calculated using the *glmer* function in the *lme4* package (Bates et al., 2015) in R. For species whose optimal models included a month or lunar brightness term, we constructed 95% prediction intervals using 1,000 bootstrapping iterations with the *bootMer* function in the *lme4* (Bates et al., 2015) package in R. For purposes of display of the prediction intervals, the values of all other covariates in optimal models were set to the mean, and the mineral lick chosen to represent the model results was the lick associated with the median random intercept value.

## 3 RESULTS

### 3.1 Camera trapping

Over all rotations, the average number of camera traps placed in each mineral lick was 1.2 cameras, with a range of 1–3 cameras per lick. Camera traps captured a total of 319,926 photographs over 6,255 camera nights during the study period. The number of camera nights at each mineral lick was highly variable, with a range of 10 days to 265 days, since many cameras malfunctioned, and several mineral licks flooded or experienced some disturbance (i.e., a tree falling in front of the camera). Mineral licks that had fewer than 55 camera nights of data recorded at the end of the study period were excluded from the analyses. After all empty photographs were removed, 143,497 photographs remained from 52 mineral licks. These photographs collectively described 5,210 independent visitation events by mammals and 1,264 visitation events from birds (Table 1). Seven medium‐ to large‐bodied mammal species and one large‐bodied bird species (weight > 1 kg), the blue‐throated piping guan, were recorded in more than 50 visitation events, and these species were included in regression analyses. Species richness at each mineral lick varied from 1 to 15 species of identifiable mammals and birds, with 5 species as the median and 5.83 (*SD* = 2.68) species as the mean number of species visiting a mineral lick over the duration of the study period.

### 3.2 Visit frequencies

The blue‐throated piping guan (*Pipile cumanensis*) was the most common large‐bodied bird visitor to mineral licks, with a mean visit frequency of 20.35 (95% CI 0–41.79) visits per 100 camera nights but only visited four of the sampled mineral licks (Table 1). Other common visitors included the gray‐winged trumpeter (*Psophia crepitans*), nocturnal curassow (*Nothocrax urumutum*), and Spix's guan (*Penelope jacquacu*) (Table 1).

Red brocket deer were the most frequent mammal visitors to mineral licks, with a mean visit frequency of 103.20 (95% CI 0–242.40) visits per 100 camera nights, followed by the paca and agouti with mean visit frequencies of 60.17 (95% CI 8.67–111.68) and 56.00 (95% CI 0–130.47) (Table 1). The collared peccary and tapir each had mean visit frequencies greater than 35 visits per 100 camera nights. The red howler monkey, gray brocket deer (*Mazama gouazoubira*), and Linnaeus's two‐toed sloth (*Choloepus didactylus*) were also frequent visitors, with mean visit frequencies greater than 12 visits per 100 camera nights (Table 1).

### 3.3 Activity patterns

**FIGURE 2:**
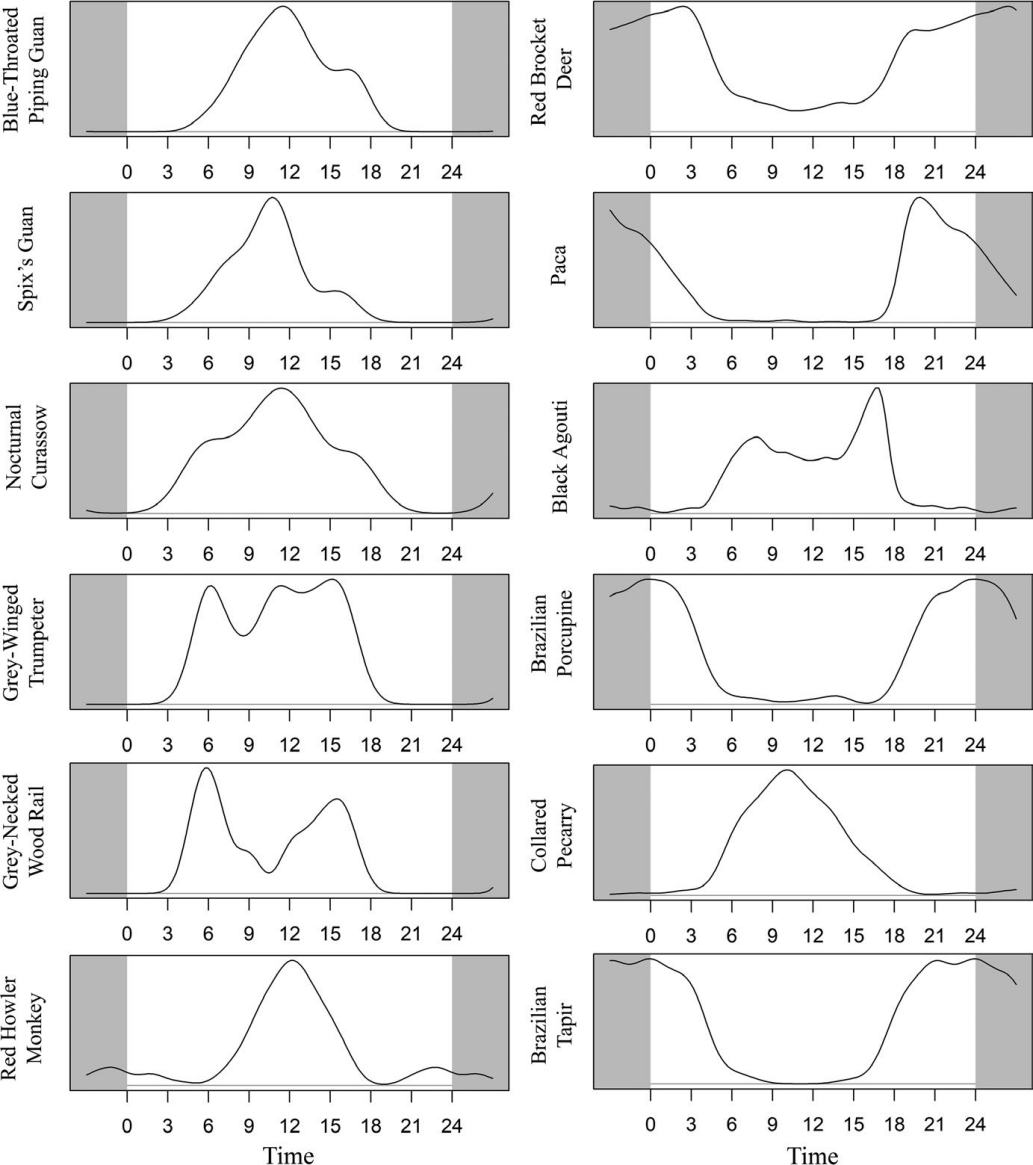
Kernel density plots of relative density of activity patterns for medium‐ and large‐bodied bird and mammal species at 52 mineral licks in the Sucusari River Basin in the northeastern Peruvian Amazon. Only bird species which were recorded more than ten times and mammal species more than 50 times are shown. Shaded regions on the margins of graphs show continuation of trends from the opposite end of the graph

Analysis of activity patterns of the blue‐throated piping guan, gray‐winged trumpeter, nocturnal curassow, Spix's guan, and gray‐necked wood rail revealed that all of these species except for the gray‐necked wood rail exhibited diurnal activity patterns (Figure 2). Activity of the blue‐throated piping guan, Spix's guan, and nocturnal curassow peaked close to 12.00 hr, while activity of the gray‐winged trumpeter remained relatively constant from 06.00 hr to 15.00 hr (Figure 2). The gray‐necked wood rail showed crepuscular activity, with a bimodal distribution peaking at 06.00 hr and 16.00 hr, close to dawn and dusk under the canopy (Figure 2).

The paca, Brazilian porcupine, and tapir exhibited nocturnal mineral lick activity patterns. Paca activity peaked at around 20.00 hr and decreased throughout the night (Figure 2). Porcupine activity peaked at midnight, while tapir visited relatively evenly throughout the night. The collared peccary and red howler monkey exhibited diurnal activity patterns, with a peak in activity at around 10.00 hr and 12.00 hr, respectively (Figure 2). The agouti showed both diurnal and crepuscular activity, with slight peaks at dawn and dusk. The red brocket deer exhibited mostly nocturnal activity, with a slight peak at 03.00 hr, but it was also active throughout the day (Figure 2).

### 3.4 Probability of recording groups

Three species of birds, the blue‐throated piping guan, nocturnal curassow, and gray‐winged trumpeter commonly visited mineral licks in groups. The blue‐throated piping guan was frequently observed either alone or in pairs, with one visit consisting of five individuals. The nocturnal curassow and gray‐winged trumpeter tended to be in pairs when visiting mineral licks. Groups of Spix's guan were recorded on several occasions, including one visit with four individuals, although groups of two or more were recorded on 13.04% of visits.

Collared peccaries and red howler monkeys frequently visited mineral licks in groups, with minimum group sizes up to 11 individuals for the collared peccary and 5 individuals for the red howler monkey. The black agouti and Brazilian porcupine visited mineral licks alone most of the time, but minimum group sizes of 2 or 3 individuals were recorded 35 times (4.01% of visits) and 60 times (9.54% of visits), respectively. Only one individual was recorded in most visits by red brocket deer, tapir, and paca, but pairs of red brocket deer were recorded 59 times (3.31% of visits), pairs of tapirs 17 times (4.40% of visits), and pairs of paca 56 times (6.01% of visits).

### 3.5 Seasonality of mineral lick visitation

**FIGURE 3:**
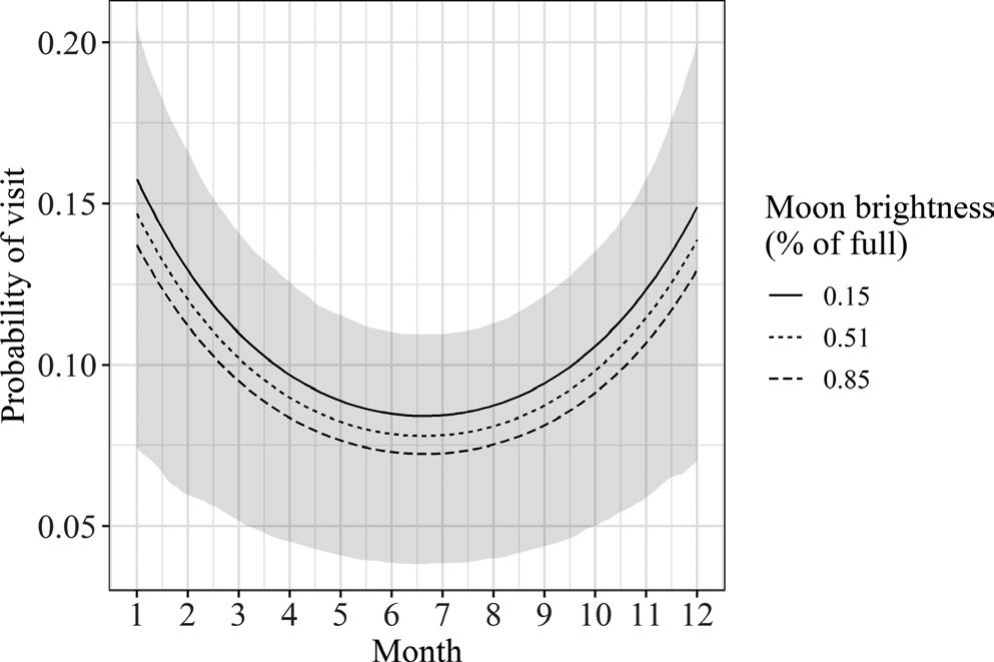
Generalized linear mixed‐effects model results showing seasonal and lunar trends in mineral lick visitation for the red brocket deer (*Mazama americana*) at 52 mineral licks in the Peruvian Amazon. Shaded area shows bootstrap prediction interval calculated using the mean values of all relevant covariates except for month

Six of the eight species analyzed exhibited seasonal mineral lick visitation and visitation was related to the brightness of the moon for three species. Optimal generalized linear mixed‐effects models of mineral lick visitation showed that visitation was related to both the month and lunar phase for the red brocket deer (Table 2). Red brocket deer were most likely to visit mineral licks during the rainy season, with a peak in visitation in December and January (Figure 3). The red brocket deer was most likely to visit mineral licks on nights when the moon was closer to a new moon, with lower brightness (Figure 3).

**Table 2:** Generalized linear model results of the factors influencing mineral lick visitation for seven mammal species and one bird species at 52 mineral licks in the Peruvian Amazon

Fixed effects	Δ AIC	Weight
Blue‐Throated Piping Guan (*Pipile cumanensis*)		
*Month*	–	0.54
*Month + Lick Type*	1.51	0.25
Red Brocket Deer (*Mazama americana*)		
*Lunar + Month*	–	0.35
*Lunar + Month + Lick Type*	0.14	0.33
*Lunar + Month + Lick Type + Distance from Water*	1.48	0.17
Brazilian Tapir (*Tapirus terrestris*)		
*Elevation + Lick Size + Month*	–	0.34
*Elevation + Lick Size + Month + Lunar*	0.84	0.22
*Elevation + Lick Size + Month + Lunar + Dist from Camps*	1.24	0.18
Paca (*Cuniculus paca*)		
*Lunar + Lick Size + Slope*	–	0.50
*Lunar + Lick Size + Slope + Lick Type*	1.21	0.27
Collared Peccary (*Pecari tajacu*)		
*Lick Size + Lick Type + Month*	–	0.33
*Lick Size + Lick Type + Month + Slope*	0.25	0.29
*Lick Size + Lick Type + Month + Slope + Dist from Camps*	0.71	0.23
Brazilian Porcupine (*Coendou prehensilis*)		
*Lunar + Elevation + Dist from Camps + Lick Size + Dist from Water + Lick Type*	–	0.68
*Lunar + Elevation + Dist from Camps + Lick Size + Dist from Water + Lick Type + Slope*	1.99	0.25
Black Agouti (*Dasyprocta fuliginosa*)		
*Elevation + Lick Size + Month*	–	0.4
*Elevation + Lick Size + Month + Lick Type*	0.22	0.36
*Elevation + Lick Size + Month + Lick Type + Slope*	1.93	0.15
Red Howler Monkey (*Alouatta seniculus*)		
*Elevation + Dist from Camps + Lick Type + Month*	–	0.45
*Elevation + Dist from Camps + Lick Type + Month + Slope*	0.41	0.36

Note

Only models within 2 AIC of the optimal model are shown.

**FIGURE 4:**
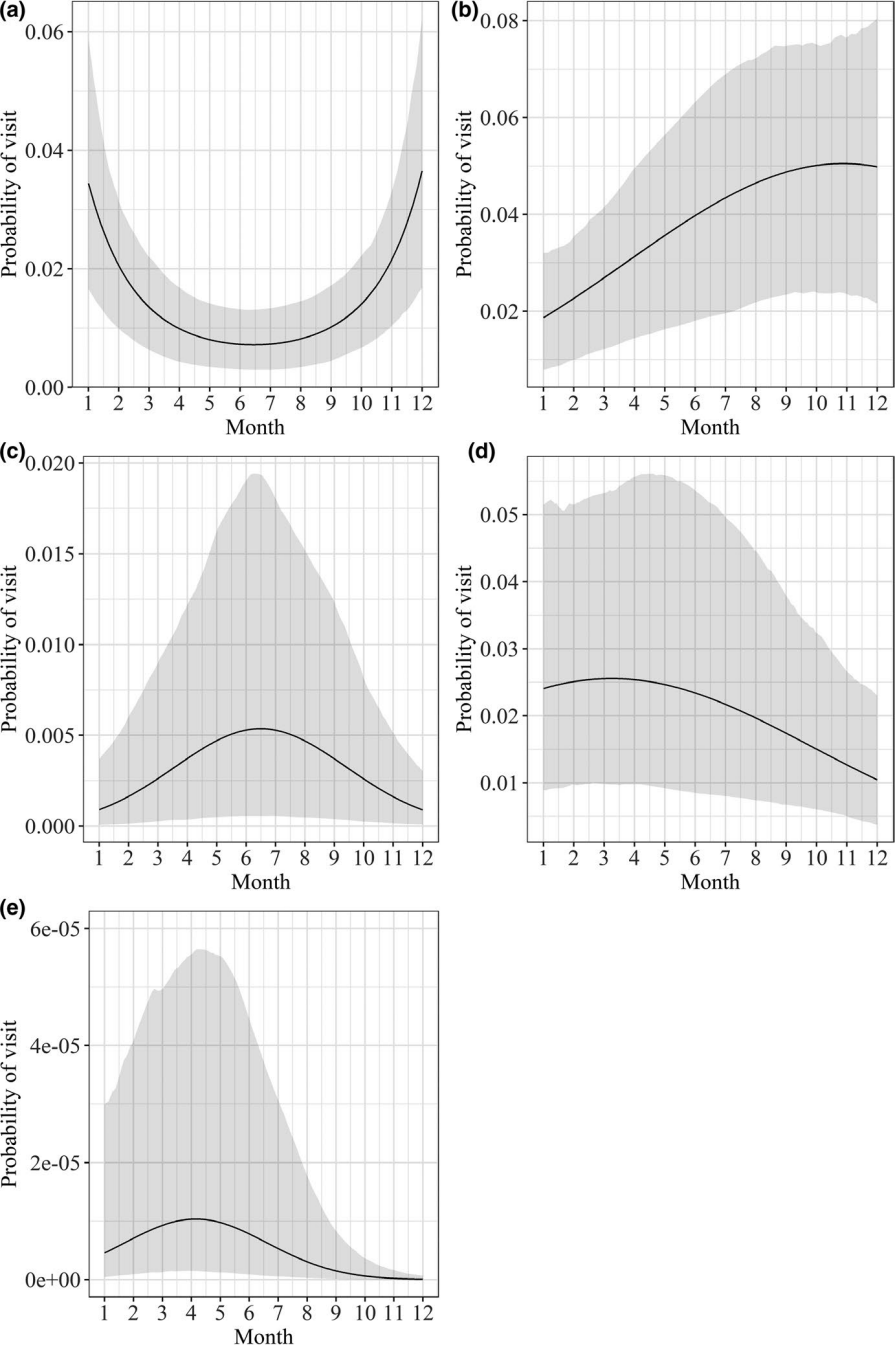
Generalized linear mixed‐effects model results showing seasonal mineral lick visitation for the (a) tapir (*Tapirus terrestris*), (b) black agouti (*Dasyprocta fuliginosa*), (c) red howler monkey (*Alouatta seniculus*), (d) collared peccary (*Pecari tajacu*), and (e) blue‐throated piping guan (*Pipile cumanensis*) at 52 mineral licks in the Peruvian Amazon. Shaded area shows bootstrap prediction interval calculated using the mean values of all relevant covariates except for month

**FIGURE 5:**
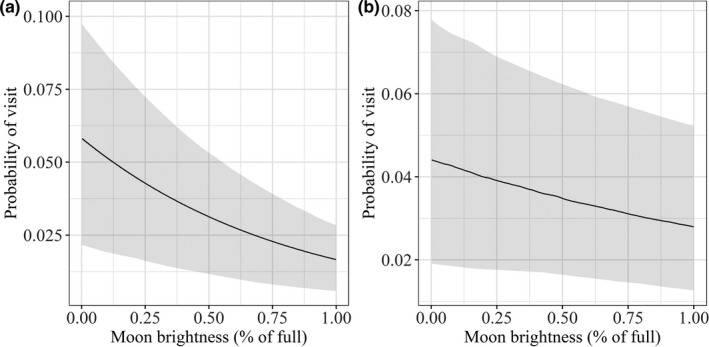
Generalized linear mixed‐effects model results showing the relationship between lunar brightness and mineral lick visitation for the (a) paca (*Cuniculus paca*) and (b) Brazilian porcupine (*Coendou prehensilis*) at 52 mineral licks in the Peruvian Amazon. Shaded area shows bootstrap prediction interval calculated using the mean values of all relevant covariates except for month

Optimal models for the tapir, black agouti, red howler monkey, collared peccary, and blue‐throated piping guan showed that visitation at mineral licks was related to the month of the year (Table 2). The tapir was most likely to visit mineral licks during the wet season, with a peak in visitation in December and January (Figure 4a). Black agouti visitation at mineral licks peaked in October and remained relatively high through December (Figure 4b). The red howler monkey was most likely to visit mineral licks in the dry season, with a peak in visitation between June and July (Figure 4c). The collared peccary showed increased visitation to mineral licks in March and April (Figure 4d). The blue‐throated piping guan also showed increased visitation in April through May (Figure 4e). Model results for the paca and Brazilian porcupine showed that mineral lick visitation was related to the lunar cycle, but not month of the year (Table 2). For both species, the probability of a visit was higher when the brightness of the moon was low (around the new moon) (Figure 5).

Several environmental covariates appeared in optimal models of visitation, but the combination of relevant covariates varied among species (Table 2) including lick size (5 species), elevation (4 species), slope (1 species), lick type (3 species), distance from hunting camps (2 species), and distance from water (1 species). Full reporting of all coefficients of optimal models can be found in Table S1.

### 3.6 Seasonality of records of groups

**FIGURE 6:**
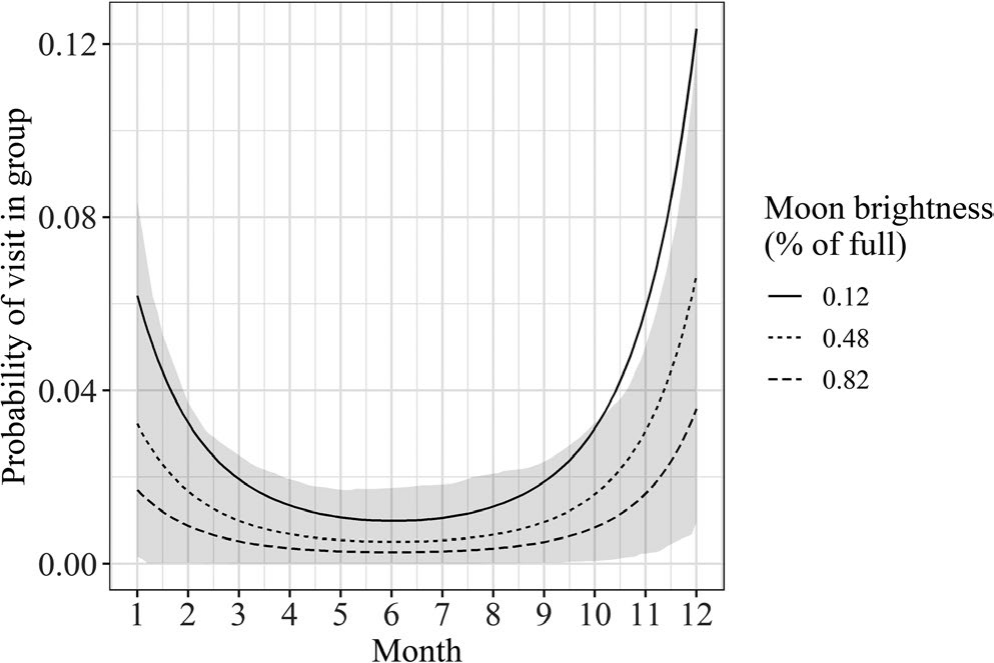
Generalized linear mixed‐effects model results showing seasonal probabilities of recording groups for the tapir (*Tapirus terrestris*) at 52 mineral licks in the Peruvian Amazon. Shaded area shows bootstrap prediction interval calculated using the mean values of all relevant covariates except for month

Only one species, the tapir, exhibited temporal variability in the probability of recording groups at mineral licks. The optimal model for the tapir included both month of the year and brightness of the moon as covariates (Table 3). Groups of tapir were most likely to be recorded during the wet season, in December and January, and when the brightness of the moon was lowest (around the new moon) (Figure 6). Optimal models of the probability of recording groups included only environmental covariates for the blue‐throated piping guan, red brocket deer, paca, collared peccary, and red howler monkey (Table 3). For the Brazilian porcupine and black agouti, the optimal model was the intercept‐only model (Table 3).

**Table 3:** Generalized linear mixed‐effects model results of the factors influencing the probability of recording groups for seven mammal species and one bird species at 52 mineral licks in the Peruvian Amazon

Fixed effects	Δ AIC	Weight
Blue‐Throated Piping Guan (*Pipile cumanensis*)		
*Slope + Dist from Water*	–	0.46
*Slope + Dist from Water + Dist from Camps*	0.21	0.42
Red Brocket Deer (*Mazama americana*)		
*Elevation + Slope + Dist from Water + Lick Type*	–	0.44
*Elevation + Slope + Dist from Water + Lick Type + Lunar*	0.76	0.3
*Elevation + Slope + Dist from Water + Lick Type + Lunar + Dist from Camps*	1.83	0.18
Brazilian Tapir (*Tapirus terrestris*)		
*Lunar + Month*	–	0.44
*Lunar + Month + Dist from Water*	0.89	0.28
Paca (*Cuniculus paca*)		
*Slope*	–	0.49
*Slope + Elevation*	0.92	0.31
Collared Peccary (*Pecari tajacu*)		
*Elevation + Lick Size*	–	0.37
*Elevation + Lick Size + Month*	0.63	0.27
*Elevation + Lick Size + Month + Lick Type*	0.98	0.23
Brazilian Porcupine (*Coendou prehensilis*)		
*Intercept Only*	–	0.43
*Lick Size*	0.78	0.29
*Lick Size + Slope*	1.77	0.18
Black Agouti (*Dasyprocta fuliginosa*)		
*Intercept Only*	–	0.42
*Month*	0.53	0.33
Red Howler Monkey (*Alouatta seniculus*)		
*Dist from Camps + Lick Size + Dist from Water*	–	0.63
*Dist from Camps + Lick Size + Dist from Water + Month*	1.96	0.23

Note

Only models within 2 AIC of the optimal model are shown.

As above, several environmental covariates appeared in optimal models of grouping, but the combination of relevant covariates varied among species (Table 3) including lick size (2 species), elevation (2 species), slope (3 species), lick type (1 species), distance from hunting camps (1 species), and distance from water (3 species). Full reporting of all coefficients of optimal models can be found in Table S2.

## 4 DISCUSSION

Our results describe new patterns associated with visitations of species at mineral licks and associations between visitations, seasons, or lunar phase for a majority of the species that frequently visit mineral licks. In addition, we describe mineral lick visitation for the nocturnal curassow, which has not previously been reported to frequent mineral licks but was recorded 24 times during our study. Our study builds upon results reported by Blake et al. (2011), who investigated patterns of visitation at four mineral licks in eastern Ecuador.

### 4.1 Activity patterns and visit frequencies

None of the species recorded visited all mineral licks in the study. For example, the red brocket deer, which was recorded during over 1,700 independent visitation events, visited 88.46% of mineral licks in the study. The red howler monkey visited only 30.77% of mineral licks. Thus, not all mineral licks may be active for all species at all times, potentially because of the mineral composition of the lick and/or the geographic location. If a species is only active at a few mineral licks, and access to those licks is limited by seasonal changes, seasonal trends would appear in model results.

Overall, 50% of the species for which activity patterns were analyzed exhibited diurnal activity, 17% exhibited crepuscular activity, and 33% exhibited nocturnal activity patterns. For the diurnal species, most activity peaked at 12.00 hr, but some variation existed between species. For the nocturnal species, there was lots of variation in activity, but most activity occurred between 20.00 hr and 03.00 hr. Our activity data for mammals including the tapir, paca, collared peccary, red howler monkeys, and red brocket deer line up with other studies from the Amazon (e.g., Blake et al., 2010, 2013; Harmsen et al., 2011; Ospina, 2011). Few studies discuss the activity patterns of the nocturnal curassow, but Parker (2002) describes the nocturnal curassow's activity as “partially diurnal,” with peaks in foraging activity just after dawn and in late afternoon, but also stated that the curassow typically hides during the middle of the day. Our data showed that nocturnal curassows in this region are almost purely diurnal, at least in regard to mineral lick visits, with a peak in activity in mineral licks at 12.00 hr rather than at dawn, dusk, or at night.

We also report several results that were not reported by Blake et al. (2011) or elsewhere. For example, Blake et al. (2011) noted that frugivorous birds, such as the common piping guan, visit mineral licks more frequently, but they did not record the nocturnal curassow and only rarely recorded the Spix's guan. We report both species more than 20 times each, but they only visited 19.23% and 21.15% of mineral licks in the study, respectively. Blake et al. (2011) and Tobler et al. (2009) both reported variation in visitation among different mineral lick sites. Our results show that to capture the full sample of variation in visitation by birds and mammals among mineral lick sites, a large sample of mineral licks is needed.

### 4.2 Lunar cycles and visitation

Visitation for three of the nocturnal species that visited the mineral licks was related to the lunar cycle. Red brocket deer, Brazilian porcupines, and paca were less likely to visit mineral licks during nights when the moon was brighter. We suggest that this decline in visitation could be due to a heightened risk of predation at mineral licks when then moon is bright, and when visibility is better for predators (Huck et al., 2017). Pratas‐Santiago et al. (2017) also showed that the activity of the paca was lowest during the bright moon phases. Wild felids such as ocelots, pumas (*Puma concolor*), and jaguars are all present in the MKRCA and were recorded visiting mineral licks periodically during this study (e.g., Griffiths et al., 2020). Predators have also been recorded at mineral licks in other regions, such as the puma and jaguar (e.g., Izawa, 1993; Matsuda & Izawa, 2008) as well as antipredator behavioral adaptations from prey species visiting mineral licks (e.g., Link & Fiore, 2013; Link et al., 2011; Ospina, 2011). The avoidance of mineral licks during the brighter moon by the red brocket deer, Brazilian porcupine, and paca may suggest that mineral licks are risky places for some species.

### 4.3 Seasonality of visitation

Mineral lick visitations by the blue‐throated piping guan, red brocket deer, tapir, red howler monkey, collared peccary, and black agouti were seasonal. Seasonal mineral lick use could be due to differential use of habitats throughout the year, particularly as access to and movement across some regions is restricted by rising waters in creeks and rivers during the rainy season. Tapirs in particular were shown by Tobler (2008) to walk over 10 km to visit mineral lick sites and actively shifted their movement to include palm swamps when the fruit of the aguaje palm (*Mauritia flexuosa*) was in season. Similarly, Sekulic (1982) showed that food resources of the red howler monkey were more patchily distributed during the dry season, which caused changes in the movement of the species. Aliaga‐Rossel (2004) found that home range sizes for the Central American agouti (*Dasyprocta punctata*) varied seasonally as well, in response to availability of fruit resources. In addition, the red brocket deer avoids flooded forest during the wet season, and those located in floodplain forest shift their diet to include woodier foods during that time due to resource scarcity (Bodmer, 1990). Similarly, gray brocket deer show seasonal changes in home range size due to seasonal scarcity of food resources (Black‐Décima, 2000).

Our results line up with those reported by Blake et al. (2011), including high frequency of lick use by red howler monkeys in the dry season and increased tapir visitation at some sites at the end of the year. The authors suggested that higher lick use by red howler monkeys in the dry season was related to a shift in diet to include a greater proportion of leaves (Blake et al., 2010), which was shown by De Souza et al. (2002) for the red‐handed howler monkey (*Alouatta belzebul*). The diet of the tapir is also made up of fruit and foliage (Montenegro, 2004) and, like the red howler monkey, mineral lick visitation was highly seasonal. In this region of Peru, a main food source for the tapir is fruit from the aguaje palm (*M. flexuosa*) (Bodmer, 1990; Virapongse et al., 2017), which dominates vast palm swamps across the MKRCA (Endress et al., 2013; Gilmore et al., 2013; Horn et al., 2011). Within the MKRCA, the aguaje palm fruits from approximately May to August (Gilmore et al., 2013). It is possible that during this time, tapirs are consuming fruit as a larger proportion of their diet and so, like howler monkeys, they visit mineral licks less frequently. While the diet of the collared peccary has not been well‐studied, the species has been known to consume fruits (Bodmer & Ward, 2006), and so seasonal peccary visitation to mineral licks may also follow the changing availability of fruit.

Blake et al. (2011) also showed a negative relationship between rainfall and visitation of the common piping guan (*Pipile pipile*), with visitation dropping in the rainy season. Here, we found a similar result for the blue‐throated piping guan, where model results showed that visitation to mineral licks rapidly increased in the months leading up to May. The breeding season for the blue‐throated piping guan in the wild is thought to be from May to November (del Hoyo, 1994), although very little is known about the piping guan's reproductive behavior (Kozlowski et al., 2018). It could be hypothesized that, in this region, blue‐throated piping guans increase their frequency of visitation to mineral licks in preparation for reproduction. A review conducted by Muñoz and Kattan (2007) described the diet of the blue‐throated piping guan as made up entirely of fruits and suggested that seasonal changes in diet are possibly due to changes in fruit availability. In this sense, our results may add evidence to that hypothesis, where blue‐throated piping guans exhibit seasonal visitation to mineral licks to make up for a lower quality or different seasonal diet, similar to that of the tapir and red howler monkey. However, since the blue‐throated piping guan only visited four mineral licks in the study, our results for seasonal visitation could be biased. For example, three of the mineral licks which experienced heavy visitation by the blue‐throated piping guan were only camera trapped from August to November. Two of these mineral licks were resampled for more data from January to April, and the last mineral lick was camera trapped from April to June. As such, if these were the only four mineral licks in the study relevant to the blue‐throated piping guan, higher probabilities of visitation during the dry season could be a relic of heavier camera trapping at the relevant mineral lick sites during that time.

Model results for the red brocket deer, collared peccary, and agouti also showed seasonal visitation, even though these species are known to breed year‐round (El Bizri et al., 2018; Mayor et al., 2011). Several other studies have described increased visitation at mineral licks before reproduction for other species, including Amazonian bats (Bravo et al., 2008; Voigt et al., 2008), white‐tailed deer (Atwood & Weeks, 2002, 2003), and African elephants (Holdø et al., 2002). Our results do not line up with those reported by Montenegro (2004), who reported no seasonality in visitation by the blue‐throated piping guan or the tapir while camera trapping at 14 mineral licks. Similarly, Link et al. (2012), who studied only two mineral licks, reported no seasonality of visitation and no relationship with the lunar cycle for the tapir and paca. We suggest that our large sample size of mineral licks allowed us to capture a fuller range of variation of visitation patterns at mineral licks.

Since our camera trap survey did not run from May to July, it is possible that crucial data was missed that could improve model fit and provide context to observed trends in seasonal visitation. For the howler monkey in particular, the peak in mineral lick visitation was predicted to occur during this period. While the addition of data from May to July would likely improve the fit of the models presented, it is likely that inferences and results would be unchanged from those presented here since our survey efforts for the rest of the year were robust and the model selection process clearly selected seasonality as an important factor in visitation.

### 4.4 Records of groups

Only tapirs were recorded in groups at mineral licks seasonally and related to the brightness of the moon. Although little is known about the reproduction of tapirs in the wild, evidence has suggested reproduction is not seasonal (Salas & Kim, 2002). Since the pairs of tapirs recorded in this study were adults, it is possible that the increase in grouping of tapirs at mineral licks during the wet season is a relic of increased visitation, where several tapirs visit at the same time. Montenegro (1998) also hypothesized that mineral licks were important sites of communication for tapir, through urine deposition.

The tapir was more likely to be recorded in groups when the moon was less bright, which could correspond to lower visitation when the moon was brighter. Even though relatively few groups of tapirs were recorded, these observations were spread among five different mineral licks and four different months of data collection. Since tapirs visit mineral licks very regularly (Tobler, 2008) and individuals could not be identified, it is possible that the same pairs of tapirs visited the same mineral lick multiple times around the new moon, skewing the model results. These findings fit with the effects of the lunar cycle on animal behavior in general as they have been well described in regard to the timing of reproduction of marine animals (e.g., Omori, 1995) and amphibians (e.g., Grant et al., 2009), activity patterns of prey species (e.g., Huck et al., 2017), and singing behavior in some bird species (e.g., York et al., 2014).

Environmental covariates were significant in both series of models of visitation for almost all species assessed. Many of these environmental covariates were habitat‐specific, such as elevation and slope, indicating that the spatial use of the landscape is a significant factor in mineral lick visitation, which has been previously suggested by Tobler et al. (2009). The significance of lick‐specific covariates, such as lick size and lick type, suggests that the physical attributes of the lick itself may provide an indicator of the quality of the lick and influence visitation. The importance of the distance from hunting camps term in the models of some species, such as the howler monkey, may indicate that hunting pressure influences mineral lick visitation, either through reduction of species abundance or behavioral adaptations to risk (Laundré et al., 2010).

## 5 CONCLUSIONS

Overall, our results showed that based on visit frequency, mineral licks are a more important ecological resource than was previously known for many understudied species of birds and mammals. Visits at these sites were linked to abiotic factors for several species, although the drivers behind the variation in visitation at mineral licks remain unknown. We conclude that further research is needed to understand the drivers of variation in mineral lick visitation and behavior at mineral licks of birds and mammals in Amazonia.

## CONFLICT OF INTEREST

The authors do not have any conflicts of interest to declare.

## AUTHOR CONTRIBUTIONS

**Brian M. Griffiths:** Conceptualization (lead); formal analysis (lead); funding acquisition (lead); investigation (lead); methodology (equal); visualization (lead); writing – original draft (lead). **Mark Bowler:** Conceptualization (equal); methodology (equal); resources (equal); supervision (equal); writing – review & editing (equal). **Michael P. Gilmore:** Conceptualization (equal); funding acquisition (equal); investigation (equal); methodology (equal); resources (equal); writing – review & editing (equal). **David Luther:** Conceptualization (equal); formal analysis (equal); supervision (equal); writing – original draft (equal); writing – review & editing (equal).

## Supporting information

Tables S1‐S2

## Data Availability

The data for this manuscript can be found on Dryad, https://doi.org/10.5061/dryad.bcc2fqzb2 The data have been embargoed for a period of one year from the publication of this manuscript.
