# Corrigendum

**DOI:** 10.1002/ece3.9316

**Published:** 2022-09-12

In the paper published by Griffiths et al. (2020), the authors would like to correct the mistake in Table 1, row 1. The total number of visitation events for the red brocket deer is 1733, and the maximum visit frequency is 205.10.

The authors apologize for this oversight.
