# Supplementary material for: Temporal patterns of visitation of birds and mammals at mineral licks in the Peruvian Amazon
Source: Ecol Evol. 2020 Nov 11;10(24):14152–64. doi: 10.1002/ece3.7006 (PMC7771178; doi:10.1002/ece3.7006)
Supplement: Supplementary file 1 — Tables S1‐S2 [file ECE3-10-14152-s001.docx]

Appendix A. Supplementary Tables

Table S1. Coefficients of optimal generalized linear model results assessing bird and mammal visitation patterns at 52 mineral licks in the Sucusari River Basin.

| **Covariate** | **Coefficient** | **Std. Error** | **Lower CI** | **Upper CI** |
| --- | --- | --- | --- | --- |
| Blue-Throated Piping Guan (*Pipile cumanensis*) | | | | |
| Month | -98.32 | 22.29 | -142.01 | -54.63 |
| Month^2^ | -56.69 | 19.76 | -95.41 | -17.96 |
| Red Brocket Deer (*Mazama americana*) | | | | |
| Lunar | -0.08 | 0.04 | -0.17 | 0.00 |
| Month | -2.33 | 3.20 | -8.61 | 3.95 |
| Month^2^ | 15.49 | 4.74 | 6.20 | 24.78 |
| Brazilian Tapir (*Tapirus terrestris*) | | | | |
| Elevation | 0.70 | 0.29 | 0.14 | 1.26 |
| Lick Size | 0.52 | 0.25 | 0.03 | 1.00 |
| Month | -0.32 | 5.12 | -10.36 | 9.71 |
| Month^2^ | 37.12 | 8.19 | 21.07 | 53.17 |
| Paca (*Cuniculus paca*) | | | | |
| Lunar | -0.46 | 0.06 | -0.57 | -0.35 |
| Lick Size | -1.20 | 0.44 | -2.07 | -0.33 |
| Slope | -0.70 | 0.30 | -1.30 | -0.11 |
| Collared Peccary (*Pecari tajacu*) | | | | |
| Lick Size | -1.14 | 0.51 | -2.13 | -0.14 |
| Lick Type (mud) | -1.10 | 0.73 | -2.54 | 0.34 |
| Month | -19.84 | 5.23 | -30.09 | -9.59 |
| Month^2^ | -8.21 | 6.99 | -21.90 | 5.48 |
| Brazilian Porcupine (*Coendou prehensilis*) | | | | |
| Lunar | -0.16 | 0.06 | -0.28 | -0.05 |
| Elevation | -0.71 | 0.35 | -1.40 | -0.02 |
| Dist from Camps | 0.55 | 0.30 | -0.04 | 1.13 |
| Lick Size | 0.49 | 0.30 | -0.11 | 1.09 |
| Dist from Water | 0.61 | 0.36 | -0.09 | 1.31 |
| Lick Type (mud) | -1.98 | 0.78 | -3.51 | -0.46 |
| Black Agouti (*Dasyprocta fuliginosa*) | | | | |
| Elevation | -0.44 | 0.27 | -0.97 | 0.09 |
| Lick Size | -1.07 | 0.38 | -1.83 | -0.32 |
| Month | 24.51 | 4.46 | 15.76 | 33.26 |
| Month^2^ | -7.29 | 5.29 | -17.66 | 3.08 |
| Red Howler Monkey (*Alouatta seniculus*) | | | | |
| Elevation | -0.84 | 0.50 | -1.81 | 0.14 |
| Dist from Camps | 0.74 | 0.43 | -0.10 | 1.57 |
| Lick Type (mud) | -2.72 | 1.24 | -5.14 | -0.29 |
| Month | 1.65 | 13.05 | -23.93 | 27.23 |
| Month^2^ | -40.77 | 12.68 | -65.63 | -15.91 |

Note: Continuous covariates are scaled

Table S2. Coefficients of optimal generalized linear mixed-effects model results assessing the probability of recording groups of tapir (*Tapirus terrestris*) at 52 mineral licks in the Sucusari River Basin.

| **Covariate** | **Coefficient** | **Std. Error** | **Lower CI** | **Upper CI** |
| --- | --- | --- | --- | --- |
| Blue-Throated Piping Guan (*Pipile cumanensis*) | | | | |
| Slope | 0.35 | 0.14 | 0.082 | 0.62 |
| Dist from Water | 0.36 | 0.21 | -0.059 | 0.78 |
| Red Brocket Deer (*Mazama americana*) | | | | |
| Elevation | 0.42 | 0.14 | 0.14 | 0.69 |
| Slope | -0.56 | 0.26 | -1.06 | -0.054 |
| Dist from Water | 0.35 | 0.18 | -0.013 | 0.703 |
| Lick Type (mud) | 1.26 | 0.51 | 0.27 | 2.25 |
| Brazilian Tapir (*Tapirus terrestris*) | | | | |
| Lunar | -0.68 | 0.35 | -1.36 | 0.0026 |
| Month | 4.67 | 5.39 | -5.90 | 15.24 |
| Month^2^ | 13.91 | 6.56 | 1.05 | 26.78 |
| Paca (*Cuniculus paca*) | | | | |
| Slope | -0.55 | 0.40 | -1.33 | 0.23 |
| Collared Peccary (*Pecari tajacu*) | | | | |
| Elevation | 0.16 | 0.058 | 0.043 | 0.27 |
| Lick Size | -0.35 | 0.18 | -0.19 | 0.50 |
| Red Howler Monkey (*Alouatta seniculus*) | | | | |
| Dist from Water | -0.12 | 0.075 | -0.26 | 0.031 |
| Dist from Camps | 0.17 | 0.081 | 0.014 | 0.33 |
| Lick Size | -0.15 | 0.11 | -0.37 | 0.06 |

Note: Continuous covariates are scaled
